# Multiple developmental pathways in organisms with developmentally complex life cycles

**DOI:** 10.3389/fcell.2025.1585073

**Published:** 2025-05-14

**Authors:** Giuseppe Fusco, Alessandro Minelli

**Affiliations:** Department of Biology, University of Padova, Padova, Italy

**Keywords:** alternation of generations, asexual reproduction, gene expression, morphogenesis, parthenogenesis, plasticity, regeneration, sexual reproduction

## Abstract

One aspect under which an organism’s life cycle can be considered complex is when the very same organism can undertake, or obligatorily undertakes, multiple developmental pathways. Examples are organisms with alternation of generations, like most plants, or organisms with reproductive and/or developmental options, like many marine invertebrates. With a broad taxonomic coverage across the eukaryotes, we survey these developmentally complex life cycles, presenting selected case studies to illustrate the relationships between the diverse developmental pathways within the same organism for what concerns morphogenesis and gene expression. We highlight the deep connections between the different types of cycles and show their relationship with phenotypic plasticity, sexual dimorphism and ecological adaptation. The collected materials and organized concepts can provide the basis for future investigations on the disparity of complex life cycles and their evolution across the tree of life.

## 1 Introduction

Elements contributing to qualify a cycle as complex have been variably articulated by different authors, generally referring to the occurrence of multiple “phases’” but with different specifications of what these “phases” are and how these are interrelated within the cycle. With different formulations, a complex life cycle has been described as one that includes abrupt ontogenetic changes in an individual’s morphology, physiology, or behaviour, usually associated with a change in habitat (e.g., Wilbur, 1980), thus passing through two or more distinct ecological and morphological phases for each complete generation (e.g., Slade and Wassersug, 1975). These definitions do not explicitly include the complexity related to the possibility of multiple generations within the same cycle, but other definitions take this aspect into account, specifying that the two or more discrete phases we can identify in a complex life cycle can be either phases in the development of an individual or distinct generations in a multiple-generation cycle (e.g., Godfrey-Smith, 2016).

In this review we concentrate on a particular way in which an organism’s life cycle can be considered complex, that is when the very same organism can undertake, or obligatorily undertakes, multiple developmental pathways. This is, for instance, the case for organisms with alternation of generations, like most plants, or organisms with reproductive and/or developmental options, like many marine invertebrates. In all these cases, multiple developmental processes are subtended by the same genome. Contra Lueth and Reski (2023), this is not an exclusive feature of plants with an alternation of generations between the haploid gametophyte and the diploid sporophyte (haplodiplontic life cycle), but a feature widespread across the whole eukaryote tree, although under different forms.

We will survey these *developmentally complex life cycles*, presenting selected case studies to illustrate the relationships between the diverse developmental pathways within the same organism for what concerns morphogenesis and gene expression. We will highlight the deep connections between the different types of cycles and show their relationship with phenotypic plasticity, sexual dimorphism and ecological adaptation.

As a terminological note, in the biological literature, “organism” is used to indicate both a species and an individual. We will use this term with the former meaning, while using “individual” or “individual organism” for the latter. The life cycle is a feature of the organism, and in organisms with multigenerational life cycles (see below), two or more individual organisms, each with its individual ontogeny and possibly with different organizational forms, are sequentially required to complete the cycle.

## 2 An overview of the different types of developmentally complex life cycles

The life cycle of an organism is the totality of transformational processes and reproductive events which, starting from a convenient life stage, can lead to the same stage in a next generation of the same organism. From zygote to zygote, but also from spore to spore, from adult to adult, or from embryo to embryo. As such, a life cycle includes development as a part of it, but can be composed of multiple developmental and reproductive phases.

For our argument, it is convenient to distinguish two main kinds of life cycles (Fusco and Minelli, 2019). In a *monogenerational life cycle*, e.g., of an earthworm, the same developmental phase (e.g., the juvenile) of the single organizational form of the organism (here, the vermiform animal) is repeated after one generation. In contrast, in a *multigenerational life cycle*, e.g., of a fern, the organism passes through a given developmental stage (e.g., the full-formed leafy plant) of a given organizational form (in this case, the sporophyte) obligatorily running over more than one generation. In the case of ferns, the daughter plants of a sporophyte are not also sporophytes, but gametophytes generated through spores, which, in turn, will produce the next sporophyte generation through gametes. In multigenerational life cycles there are reproductive phases where offspring are generated that are not of the same kind (of the same organizational form) as the parent(s), so that more than one generation is needed to return to the starting form. Multigenerational cycles are called, in a broad sense, cycles with alternation of generations.

**FIGURE 1:**
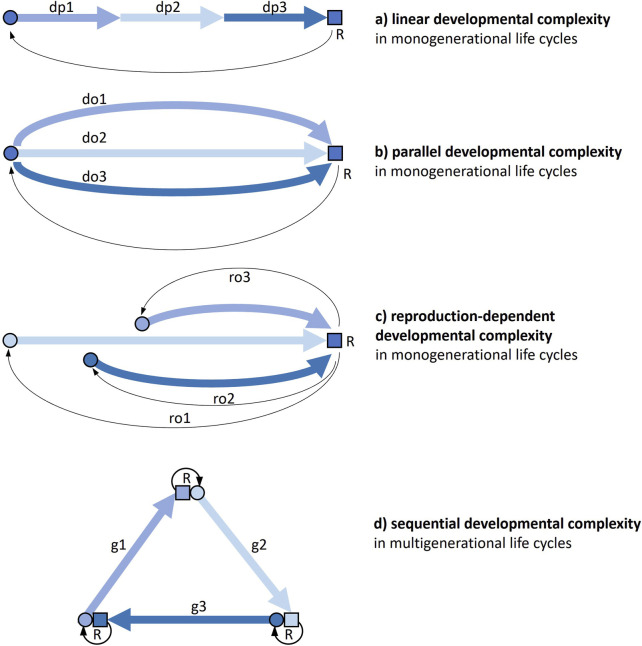
The different ways a life cycle can present developmental complexity. Thick arrows are developmental pathways, thin arrows connect parents to offspring through reproduction. **(a)** Monogenerational life cycle where individual development includes profound phenotypic transformations (e.g., holometabolous insects). **(b)** Monogenerational life cycle where alternative (facultative) options for development can be taken, so that developmental processes first diverge and then converge again in a subsequent stage (e.g., some nematodes). **(c)** Monogenerational life cycle where alternative (facultative) options for reproduction (e.g., sexual, asexual) can be taken, so that developmental processes have a different start and pathway (e.g., many plants). **(d)** Multigenerational life cycle where the cycle obligatorily passes through more than one organizational form, each with its development and reproduction (e.g., medusozoan cnidarians). Combinations of the four kinds of complexity and further developmental complications are possible. Circle, young: square, adult; do, developmental option; dp, developmental phase; g, generation; ro, reproductive option; R, reproduction.

There are different ways in which a life cycle, either monogenerational or multigenerational, can present developmental complexity (Figure 1).

A first kind of life-cycle developmental complexity (Figure 1a) is a function of the number and magnitude of changes an individual organism undergoes throughout its development. This *linear developmental complexity* is widespread across the tree of life. Just think of the contrast between embryonic and postembryonic development in many multicellular organisms. But very different developmental stages can succeed each other also along post-embryonic life. In many animals, including numerous marine invertebrates and most holometabolous insects, the adult form is markedly distinct from juvenile (larval) form(s), to the point that the passage between these segments of life is generally qualified as a metamorphosis. This kind of complexity is widespread and largely acknowledged, and we will not treat it here. However, we will return to it through the important connections this has with other types of developmental complexity (see Section 4).

In a second kind of life-cycle developmental complexity (Figure 1b), at certain stages of a monogenerational cycle, alternative options can be taken: developmental pathways can first diverge and then converge again in a subsequent stage, so that the latter can be reached through alternative paths within the same cycle. An example of this *parallel developmental complexity* is offered by some species of soil nematodes, where adverse environmental conditions trigger post-embryonic development towards the production of a so-called dauer stage, which is able to survive long periods of starvation, as an alternative to a normal third-stage juvenile.

A third type of developmental complexity (Figure 1c) is found in cycles with reproductive (rather than developmental) options, that can actually entail more individual developments, when, for instance, asexual reproduction is facultative and development has a very different start than from a fertilized egg. An example of this *reproduction-dependent developmental complexity* is the contrast between development starting from a fertilised egg and development starting from a bud in a hydra polyp.

A fourth type of developmental complexity (Figure 1d) is offered by multigenerational life cycles. This *sequential developmental complexity* can be found in many cycles with alternations of generations, haploid and diploid as in most plants, but also sexual and asexual as in many cnidarians, or unicellular and multicellular as in slime moulds.

Combinations of these four kinds of complexity are not only possible, but widespread. The midge *Heteropeza pygmaea* presents linear developmental complexity, as all holometabolous insects, but can also switch between reproducing when adult and reproducing when still a larva (paedogenesis), depending on food availability (reproduction-dependent developmental complexity). Many cnidarians have multigenerational life cycles with alternating polyp and medusa generations (sequential developmental complexity), but some species can also generate polyps from polyps and/or medusae from medusae asexually, as a reproductive option (reproduction-dependent developmental complexity).

All these cycles with multiple developmental processes qualify as “complex” life cycles, but not all complex life cycles necessarily entail markedly distinct developmental processes. For example, multigenerational life cycles with a single mode of development are found in many organisms with alternation of amphigonic and parthenogenetic generations, where development similarly starts from a laid egg, either fertilized or unfertilized, as in many (oviparous) aphids. In certain algae, similar developmental pathways, starting from a haploid spore or from a diploid zygote, take to morphologically indistinguishable gametophyte and sporophyte, respectively (haplodiplontic cycles with isomorphic generations, as in the brown alga *Ectocarpus*). These cycles are not on focus in the present review.

The idea of multiple development naturally extends to all cases of plasticity in monogenerational cycles, like caste polyphenism, ecomorphosis, cyclomorphosis and environmental sex determination. Although these phenomena are not neatly distinct from the forms of developmental complexity listed above, and some forms of plasticity are actually part of them, as we will see, in order to contain the subject of the present review within reasonable boundaries, we will leave these cycles out of the main discussion as well. A scheme of the types of developmental complexity covered by the present paper is outlined in Table 1.

**TABLE 1:** Synopsis of the life cycles with developmental complexity discussed in the present paper.

Phenomenon	Example
Parallel developmental complexity	*Caenorhabditis elegans* (nematodes)
Reproduction-dependent developmental complexity	*Kalanchoe pinnata* (angiosperms)
Sequential developmental complexity	
• Haplodiplontic cycles with heteromorphic generations	*Physcomitrium patens* (bryophytes)
• Metagenetic cycles	*Clytia hemisphaerica* (cnidarians)
• Heterogonic cycles	*Acyrthosiphon pisum* (insects)
• Alternation of solitary and colonial generations	*Botryllus schlosseri* (tunicates)
• Alternation of unicellular and multicellular generations	*Dictyostelium discoideum* (amoebozoans)

## 3 A survey of developmentally complex life cycles

### 3.1 Parallel developmental complexity

In these generally monogenerational cycles, the adult stage can be reached through alternative pathways, so that different forms of embryo, larva or juvenile eventually give rise to identical adults.

This phenomenon, known as *poecilogony*, is a form of developmental plasticity and has been recorded from insects, nematodes, polychaetes, gastropods and amphibians (Minelli and Fusco, 2010). In marine invertebrates, poecilogony generally takes the form of an option between feeding and non-feeding larvae. In the marine gastropod *Alderia modesta* the progeny from a single parent can be all planktotrophic, all lecithotrophic, or, sometimes, mixed (Krug, 1998). In some amphibians, poecilogony occurs through a form of larval resource polyphenism (Pfennig, 2021). In still other groups (e.g., in nematodes), a specific (often resting) stage is optionally inserted into an otherwise “standard” developmental sequence.

**FIGURE 2:**
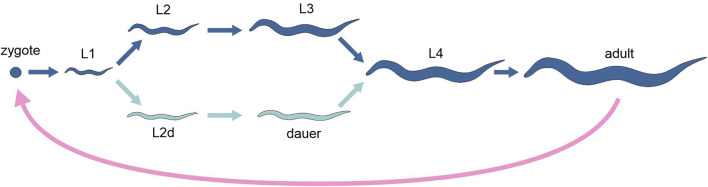
Schematic representation of the life cycle of the nematode *Caenorhabditis elegans*, highlighting the two alternative developmental options (different tones of blue). Pink arrow, sexual reproduction; light and dark blue arrows, development.

Alternative developmental pathways occurring in one and the same organism have been intensively investigated in a few species of nematodes. Under favourable conditions, the model species *Caenorhabditis elegans* develops through four juvenile stages (often called “larvae,” L1 through L4) to reach the adult stage in 3 days at 20°C. Conversely, under unfavourable conditions (high population density, food shortage, high temperatures), the third stage occurs in an alternative resting phenotype, called dauer, which can last up to 6 months, while the entire life span of the worm under normal conditions is 3 weeks (Figure 2). The dauer stage is highly resistant to stress and presents specific morphological, physiological and behavioural features that allow them to survive and disperse. Natural populations of *C. elegans* and other nematodes, adapted to different habitats and ecological niches, show sizable variation in the way dauer stage is induced, and genetic studies have identified numerous quantitative trait loci (QTL) associated with these differences (Billard et al., 2020).

The dauer developmental decision occurs at late stage L1 (Eisenmann, 2005). Relative to active stages, dauers appear to be transcriptionally quiescent, but show high levels of mRNA encoding the heat shock protein Hsp90, as well as elevated superoxide dismutase and catalase activities. This is consistent with the fact that dauers are able to resist metabolic stress and with the long duration of the stage. Gene expression profiles have been generated to identify gene expression changes correlated with dauer arrest and exit from arrest. Jones et al. (2001) identified 358 dauer-specific and 533 mixed-stage-specific transcripts of dauer arrest, while Wang and Kim (2003) found 2430 genes that change expression during dauer exit.

In other nematodes, developmental stages equivalent to the dauer stage of free-living species have turned into infesting parasitic stages. Under favourable conditions, *Bursaphelenchus xylophilus* multiplies via a so called “propagative cycle” through four juvenile stages (L1-L4) by feeding on plant cells (“phytophagous phase”) or fungal hyphae (“mycetophagous phase”). Under unfavourable conditions, the animal switches to the “dispersal cycle.” Here, the second-stage juvenile (L2) develops into a dispersal third-stage juvenile (D3) and subsequently, in the presence of a vector beetle, into a fourth-stage dispersal juvenile (D4). The D4 nematode is taken by the beetle to a new host tree, where the worm completes the cycle. Comparative transcriptome analysis using RNA-seq from all developmental stages of *B. xylophilus* showed that more than 9000 genes are differentially expressed in at least one stage of the cycle, including genes involved in reproduction and moulting, but also genes associated to parasitism. The dispersal-stage transcriptome showed a certain analogy to *C. elegans* dauer and revealed the distinct roles of the two dispersal stages (D3 and D4) with regards to survival and transmission (Tanaka et al., 2019).

Still other nematodes, in the genus *Strongyloides* and closely related genera, can develop either (i) by a “direct route,” by producing larvae that infect new hosts, or (ii) by an “indirect route,” with a generation of infective larvae alternating with one (*Strongyloides*) or more (*Parastrongyloides*) fully free-living generations (Viney and Morris, 2022), a phenomenon which presents affinities with dauer induction (Wang et al., 2021). *Strongyloides* spp. are obligate gastrointestinal parasites of humans and other animals, with the parasitic phase in the life cycle consisting of adult females that reproduce by mitotic parthenogenesis. The parasitic females’ progeny develops in the environment, either into infective third-stage juveniles that can infect new hosts (direct route), or into adult free-living male and female worms, that reproduce sexually in the environment and whose progeny develop into third-stage juveniles (indirect route). The switch is controlled by the environmental conditions to which the developing juveniles are exposed. Hunt et al. (2018) analysed the transcriptome of four species in the genus *Strongyloides* comparatively, focussing on genes that are differentially expressed in parasitic and free-living stages. RNA-seq data revealed diverse gene families which are uniquely upregulated in the parasitic stage of all four *Strongyloides* species, including key protein-coding gene families with a putative role in parasitism, showing some diversification of the molecular machinery involved in the parasitic life among the species.

In some tube-dwelling spionid polychaetes, both planktotrophic and lecithotrophic larvae may occur in a single species. The transcriptome of both planktotrophic and lecithotrophic developmental modes has been profiled in *Streblospio benedicti* (Marsh and Fielman, 2005). Analysis showed a more complex gene expression profile and a higher level of individual variation in expression patterns in planktotrophic larvae. At molecular level, larval developmental mode is determined by a shift in the expression patterns of a small set of genes, rather than through mode-specific regulation of largely different sets of genes.

Cycles with parallel developmental complexity bring to light at least two general questions. One is the contrast between the cycle of *Caenorhabditis*, where it is the developing juvenile that senses the environmental conditions that eventually may take to the alternative developmental route through the resting dauer stage, and the cycle of *Streblospio* (but also other polychaetes, like *Boccardia proboscidea*; Gibson and Gibson, 2004), where alternative developmental pathways are partially under maternal control, through the parental resource allocation in the egg. This contrast, rather than suggesting a further splitting in the classification of life cycles, matches with a more general phenomenon in development, where the boundary between the developmental processes under the control of the developing individual and those under the control of the mother, either (epi)genetically or physiologically, can be set at different places, with variation both within species and among closely related species. In a following section we will see this phenomenon occurring in flowering plants, with the gametophyte developing protected by the parental sporophyte. A second question is the difficulty of tracing a neat boundary with other phenomena of multiple development. One grey zone is at the boundary with sequential developmental complexity, as exemplified by the cycle of *Strongyloides*, where the switch between alternative developmental pathways takes also the value of a switch between a monogenerational and a multigenerational life cycle. Another is at the boundary with phenotypic plasticity leading to adult polyphenism, which we stipulated to leave out of this survey (Fusco and Minelli, 2010). In the polychaete *Streblospio*, the specific developmental pathway followed by the larva can lead to distinct types of adults, differing for some life-history traits, like expected lifespan or age at first reproduction (Chia et al., 1996).

### 3.2 Reproduction-dependent developmental complexity

Cycles with reproductive options are a wide class of phenomena, including both monogenerational and multigenerational cycles. Reproductive options occur whenever a given reproductive modality is facultative or optional, rather than obligate or constitutive. Parthenogenesis is facultative in many molluscs, annelids and arthropods, and also in some vertebrates, including the Komodo dragon (*Varanus komodoensis*). Self-fertilization is facultative in various hermaphrodite animals, including some pulmonate gastropods (e.g., land snails of the genus *Rumina*), while self-pollination (genetically equivalent to self-fertilization) is facultative in a number of flowering plants, including various members of the legume, orchid and aster families. Likewise, asexual reproduction is facultative in many organisms that usually reproduce sexually. In some animals, reproduction can be carried out by both the adult and a juvenile stage (paedogenesis), as the larvae or pupae of some insects.

However, not all cycles with reproductive options entail multiple developmental pathways. For instance, it is to be expected that in cycles where parthenogenesis (as an alternative to amphigony) or self-fertilization (as an alternative to cross-fertilization) are facultative, development can have an equivalent start and follow similar (or nearly identical) pathways upon one or the other type of reproduction. The same is possibly the case where asexual reproduction is through mitospores (spores that are not the result of meiosis, as it occurs in many algae and fungi), so that development start equivalently from a unicellular condition, just like from a zygote.

Restricting this form of reproductive plasticity to cases where different kinds of reproduction obligatorily bring about different starts of developmental pathways, this phenomenon involves nonetheless a vast array of multicellular organisms, distributed throughout the tree of life, which can facultatively reproduce asexually starting from a multicellular propagule. This includes diverse forms of asexual propagation by fragmentation in many fungi, algae and land plants, and all the specialized forms of vegetative reproduction in plants, like those through bulbs, bulbils, stolons, rhizomes, and tubers. In animals, asexual reproduction, when distinct from parthenogenesis, is almost always of the polycytogenous type (i.e., based on a multicellular propagule; Fusco and Minelli, 2019), through a variety of modes, including fission, strobilation and budding.

Because of the many disparate forms through which reproduction-dependent developmental complexity can occur, no general features are to be expected as to the way multiple developmental pathways are undertaken and/or regulated in different organisms. Here we only report on a sample of case studies that illustrate the state of the art in annelids and in flowering plants.

In annelids, as well as in other animals that reproduce asexually by fission, two main modes can be distinguished. In *paratomy*, division follows morphogenesis, so that new complete individuals are recognizable before their detachment from the parent. On the opposite, in *architomy* morphogenesis follows division, and the regrowth of the missing parts involves reorganization, de-differentiation and new differentiation of the fragments’ tissues. Not surprisingly, fission in metazoans is generally associated with high regenerative capacities (see also Section 3.3.4), and the relationship between reproduction and regeneration is a key aspect of the life cycles with reproduction-dependent developmental complexity.

Many annelids with high regenerative abilities practice asexual reproduction. An evolutionary connection between regeneration and asexual reproduction is suggested by the extensive similarities between the developmental mechanisms underlying these two processes. Anterior and posterior regeneration are ancestral in this phylum, supporting the hypothesis that regenerative ability is a prerequisite for fission evolution (Zattara and Bely, 2016). Fission and regeneration, although very similar in many respects, present nonetheless important differences in the extent and timing of tissue remodelling, as well as gene expression. Thus, although regeneration and asexual reproduction appear to be evolutionarily related, they do not define equivalent developmental trajectories (Zattara and Bely, 2011). In annelids, regeneration involves up- or downregulation of a large and diverse set of genes, including genes that have specific functions in development: among them, Hox genes and genes encoding factors related to nervous system patterning, and genes typically expressed in stem cells (reviewed in Kostyuchenko and Kozin, 2021).

A comparison of gene expression profiles during regular growth and regeneration (both anterior and posterior) of two species of syllids (*Sphaerosyllis hystrix* and *Syllis gracilis*) with different regenerative capacities revealed differential expression of a high number of genes (Ribeiro et al., 2019). In both species, posterior regeneration shows no major differences in gene expression with respect to the normal postembryonic process of growth, whereas anterior regeneration exhibits a markedly different pattern of gene expression. Among the upregulated genes, there are putative homologs of regeneration-related genes involved in stem-cellness and cellular proliferation, in the establishment of a new body axis and in nervous system development.

Ever since the earliest stages of these processes, regeneration and asexual reproduction are accompanied by a repatterning of the systems of positional information and changes in the molecular profile of cells. For example, in the paratomy splitting zone or at a wound site, genes of germ and multipotent cells are upregulated before the appearance of the first signs of proliferation. Early activation of the expression of the genes encoding many transcription factors is observed. All these events proceed in parallel with the repatterning of the nervous system and the molecular identity of body parts, with the involvement of Hox genes and other homeobox-containing genes (Kostyuchenko and Kozin, 2020).

In flowering plants, asexual reproduction and regeneration are no less closely linked than in animals. Somatic embryogenesis (the formation of an embryo from a somatic cell, or from a group of somatic cells) and plant regeneration both involve the developmental reprogramming of somatic cells toward embryogenesis and organogenesis, and form the basis of asexual reproduction. A series of cellular processes and molecular events mark these processes, such as somatic dedifferentiation, cell division initiation, gene expression pattern reprogramming and changes in metabolism (Guo et al., 2022). The main sources of undifferentiated stem cells are the shoot apical meristem and the root apical meristem, but plants have also secondary stem cell niches, such as the lateral meristems in the axils of leaves, which are established post-embryonically. Presence and maintenance of stem cells in secondary niches results from the reacquisition of stem cell identity by groups of cells, controlled by a complex genetic and molecular network involving transcriptional regulators, hormones and mobile signals produced by neighbouring cells (Jácome-Blásquez and Kim, 2023).

In the angiosperm model species *Arabidopsis thaliana*, somatic embryogenesis can be induced by the ectopic expression of embryo and meristem identity genes, like *LEAFY COTYLEDON1* (*LEC1*), *LEAFY COTYLEDON2* (*LEC2*) and *BABY BOOM* (*BBM*) (Horstman et al., 2017). These factors can promote somatic embryo development through a complex network of cross-regulating interactions with other transcription factors, including the MADS-domain AGAMOUS-Like15 (AGL15), hormones, and epigenetic modifiers (Joshi et al., 2022).

**FIGURE 3:**
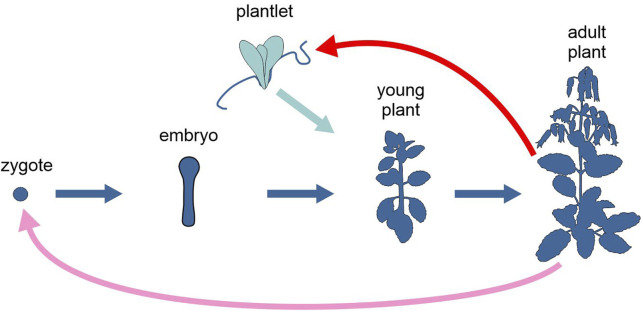
Schematic representation of the life cycle of the angiosperm *Kalanchoe pinnata*, highlighting the two alternative developmental options, upon sexual or asexual reproduction (different tones of blue). Pink arrow, sexual reproduction; red arrow, asexual reproduction; light and dark blue arrows, development. Figure not to scale.

Several species of the genus *Kalanchoe*, a group of tropical, succulent plants in the family Crassulaceae, reproduce asexually by forming plantlets on the leaf margin, either before or after leaf detachment, depending on the species (Figure 3). In *Kalanchoe pinnata,* meristem genes, such as *SHOOT MERISTEMLESS* (*STM*), are co-opted to the leaf margin, where they are differentially expressed and appear to be involved in plantlet formation (Jácome-Blásquez and Kim, 2023).

DNA methylation varies significantly across the plant life cycle, but is efficiently reinforced during reproduction, ensuring stable silencing of transposable elements. By reviewing recent findings obtained using model plant species, particularly *Arabidopsis*, rice and maize, Ibañez and Quadrana (2023) argued that since most epigenetic reinforcement appears to occur during seed formation, clonally propagated plants are expected to be hypomethylated and to undergo frequent stochastic epigenetic changes, compared to plants from both normal sexual reproduction and apomixis (i.e., in plants, clonal reproduction through seeds). In embryogenesis of both sexual and apomictic seeds, DNA methylation reinforcement by endosperm-derived short interfering RNAs (siRNAS; Ji et al., 2019) is expected to occur. Conversely, in clonal propagation, where most steps of sexual reproduction, including meiosis, fertilization, and embryogenesis, are skipped, there is a deficient, or even absent, reinforcement of DNA methylation, potentially fostering genetic differentiation through asexual propagation (Stroud et al., 2013).

Sexual and asexual reproduction can differ in their effects on the epigenome also in other ways. Zhang et al. (2023) studied how warmer temperature conditions experienced during sexual and asexual reproduction (by seeds and through stolons, respectively) affect the transcriptomes of different strawberry (*Fragaria vesca*) ecotypes. They found a more significant transcriptomic response to temperature and, for some important genes, a higher number of alternative splicing events in asexually reproducing plants than in sexually reproducing ones.

As anticipated, due to the vast disparity of modes of asexual reproduction across the eucaryote tree, reproduction-dependent developmental complexity represents a class of very diverse phenomena, with little scope for generalisation about the regulation of the different developmental pathways within the same organism. There is, however, a common thread that emerges from all the presented cases of study, which is the relationship with the regenerative processes, and the associated pattern of regulation, specification and/or maintenance of stem cells, despite the specificity of asexual/regenerative processes in each taxon. For instance, in the two annelids *S. hystrix* and *Syllis gracilis*, posterior regeneration is similar to normal growth, whereas anterior regeneration differs, which is not surprising in consideration of the fact that both postembryonic main-axis elongation and segmentation proceed in anterior to posterior direction at the rear of the body. The diversity of development within the same organism in relation to different forms of reproduction can be especially appreciated when this presents multiple modes of asexual reproduction, as in the case of vegetative reproduction and apomixis in some model angiosperms, where epigenetic signatures in apomixis are more similar to sexual reproduction than to vegetative reproduction (clonal propagation).

### 3.3 Sequential developmental complexity

#### 3.3.1 Haplodiplontic cycles with heteromorphic generations

**FIGURE 4:**
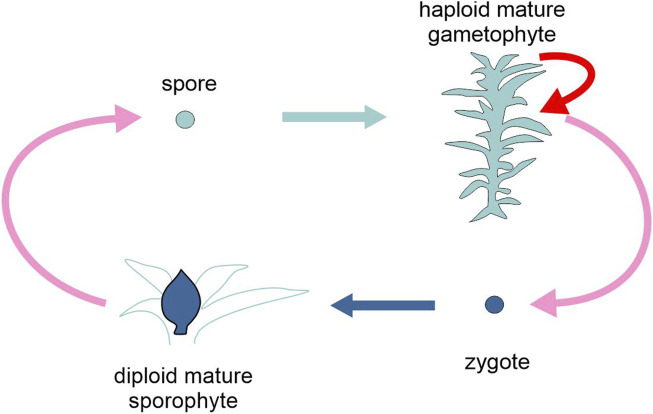
Schematic representation of the life cycle of the moss *Physcomitrium patens*, highlighting the two types of generation, gametophyte and sporophyte (different tones of blue). Pink arrows, sexual reproduction (meiosis and karyogamy); red arrow, asexual reproduction; light and dark blue arrows, development. Figure not to scale.

In these cycles, a haploid organizational form, generally indicated as the gametophyte, alternates with a diploid organizational form, generally indicated as the sporophyte. Both can reproduce sexually: the former through gametes and syngamy, the latter producing spores by meiosis. However, either or both organizational forms can also give rise to multiple asexual generations (Figure 4).

Most plants, including nearly all embryophytes (see Pelosi et al. (2023) for an exception), are haplodiplontic, as are many brown algae, some fungi and some protists. Unicellular haplodiplonts are found only among forams such as *Myxotheca*. The majority of these taxa present heteromorphic generations, i.e., there are substantial morphological differences between the two phases, which entails different developmental pathways. Among the land plants, the dominant (larger and longer-living) organizational form is the gametophyte in bryophytes, the sporophyte in tracheophytes. Within the latter group, seed plants (Spermatophyta) have extremely reduced, separate sex gametophytes, indicated as the (male) pollen grain and the (female) embryo sac. These are made up by only a few cells and are largely dependent on the parental sporophyte for nutrition and development.

In organisms with haplodiplontic cycles, each generation is regulated by a distinct developmental program that initiates either at meiosis (in the gametophyte) or at fertilization (in the sporophyte). There is a vast literature on the genetic regulation of gametophyte and sporophyte development and the transition between the two phases, in both directions (e.g., Somers and Nelms, 2023). For the scope of the present review, this information can be roughly organised around three very general questions.

A first general question is about the role of differential ploidy in determining the different developmental pathways of the (haploid) gametophyte and the (diploid) sporophyte. The following cases of study show that the link between the different ploidy level and the different organization of gametophyte and sporophyte does not seem to be necessarily strict (Minelli, 2018).

In the filamentous brown alga *Ectocarpus*, development as either a sporophyte or a gametophyte is not rigorously associated with ploidy (Cock et al., 2014). Occasionally, individual gametes have been observed to develop into haploid partheno-sporophytes (Müller, 1967) and haploid meiospores (expected to develop as gametophytes) can be induced to adopt the sporophyte developmental program by a diffusible factor produced by sporophytes (Arun et al., 2013). Still in *Ectocarpus*, specific mutations at two loci (OUROBOROS and SAMSARA) can cause the sporophyte generation to be converted into a functional gametophyte (Arun et al., 2019). Taken together, these various observations indicate that changes in ploidy are better viewed as a consequence of life cycle progression than as the determinant of sporophyte vs. gametophyte identity.

Similarly, in the moss *Physcomitrium patens*, deletions in the genes encoding the class 2 KNOTTED1-LIKE HOMEOBOX (KNOX2) transcription factors result in the development of gametophytes from diploid embryos without meiosis. This supports the hypothesis of a critical role of the evolution of KNOX2 in establishing an alternation of generations in the lineage of land plants (Sakakibara et al., 2013).

A second general question is how much the transcriptomes of the two generations differ.

Comparisons between gametophyte and sporophyte transcriptomes in mosses have revealed that gene expression is less phase-specific than in angiosperms (Sigel et al., 2018), but with significant differences between species. For example, in *Funaria hygrometrica* there is ca. 96% overlap between the two phases in the identity and expression levels of genes, and less than 1% of genes are uniquely expressed either in the sporophyte or the gametophyte, while in *P. patens* 85% of genes are expressed in both phases, and as many as 10% of genes are unique to the gametophyte (Ortiz-Ramírez et al., 2016).

The picture seems not to differ much in pteridophytes. Sigel et al. (2018) examined patterns of sporophyte (here the dominant phase) and gametophyte gene expression in the homosporous fern *Polypodium amorphum*, to assess using RNA sequencing how the common genome is expressed along the two phases. There is nearly 90% overlap in gene identity between the sporophyte and gametophyte, with less than 3% of genes uniquely expressed in either phase.

In the fern *Vandenboschia speciosa*, the overlap between gametophyte and sporophyte transcriptomes is less conspicuous, about 75%, with 1% and 23% of the transcripts expressed either in the gametophyte or in the sporophyte, respectively, but not in both (Martín-Blázquez et al., 2023). A small fraction of this phase-specific genes was annotated, but it is worth noting the presence of transcription factors, mostly involved in cell growth and differentiation, plant growth and development, as well as in stress response. Interestingly, a fraction of the specific transcripts of the sporophyte derives from transposable elements. These elements, which represent 76% of the *V. speciosa* genome, seem to have high differential activity between the two phases of the life cycle of this species.

In seed plants, the highly reduced male and female gametophytes exhibit similarly reduced transcriptome profiles, expressing fewer and different genes than sporophyte tissues (Rutley and Twell, 2015). For example, in *Arabidopsis*, only about one third of the genes expressed in the sporophyte are also expressed in pollen, and approximately 10% of pollen-expressed genes are unique to male gametophyte (Sigel et al., 2018).

Liu et al. (2023) applied comparative transcriptomics to characterize gene expression during sporogenesis and gametogenesis in *Arabidopsis*. Of a total of ∼23,000 protein-coding genes identified in RNA-seq data, 2%, 11% and 2% genes were specifically expressed in leaves (organs of the sporophyte), anthers (where male gametophytes develop) and ovules (containing the female gametophyte), respectively, while 72% genes were expressed in all three samples. Pairwise differential expression analysis between all three tissues showed that the highest number of differentially expressed genes is found in leaves with respect to both anthers (∼11,000) and ovules (∼10,000), numbers almost twice that of anthers vs. ovules, confirming the more similar expression profiles of these gametophyte containing reproductive structures.

In angiosperms, male and female gametophyte development are differentially regulated through multiple mechanisms. Beyond transcription regulation, in *Arabidopsis* both cell-type-specific alternative splicing (Misra et al., 2023) and epigenetic modifications have been reported, the latter in close connection with the alternation of generations. In *Arabidopsis* pollen development, the diploid-to-haploid transition is governed by the loss of the methylated histone peptide H3K9me2 and the demethylation of transposon-associated cis-regulatory elements. This causes important changes in chromatin accessibility and transcriptional reprogramming. The haploid-to-diploid transition is characterized instead by the global loss of H3K27me3 in the male gamete, in preparation of fertilization (Borg et al., 2021). In *Arabidopsis* ovule development, small RNAs (usually 20 to 30 nucleotides in length) control, in a sequence-specific manner, the transcriptional or post-transcriptional expression of genes (reviewed by Petrella et al., 2021).

An interesting parallel can be drawn between the so called maternal-to-zygotic transition in gene expression in animals, in which the zygotic genome is initially transcriptionally quiescent and early embryonic development is under maternal genetic control, and the diploid-to haploid transition in flowering plants, where pre-meiotic transcripts from the sporophyte persist in the haploid gametophyte, leading some spore-expressed genes to be under sporophyte control (Somers and Nelms, 2023). In maize pollen, pre-meiotic transcripts are retained for the majority of genes until ca. 11 days after meiosis. These gene products are progressively degraded and replaced with gametophyte-expressed gene products, a process which includes the activation of the haploid gametophyte genome.

A third general question is about the evolution of the genetic control of the two phases.

In both ascomycete and basidiomycete fungi and chlorophyte algae, the haploid-to-diploid transition is regulated by a pair of paralogous homeodomain protein-encoding genes. A common genetic program controlling the haploid-to-diploid transition in phylogenetically disparate eukaryotic lineages suggests that this may be the ancestral function for homeodomain proteins (Bowman et al., 2016).

In the evolution of the Viridiplantae, the specification of the diploid life stage is under the control of BELL-KNOX heterodimers. In the land plants, both BELL and KNOX genes have undergone multiple duplications, followed by a divergent association of different paralogues with either gametophyte or sporophyte development (Horst et al., 2016).

Summing up, in haplodiplontic cycles the synchronization between the alternance of the nuclear phases (haploid/diploid) and the alternance of generations (gametophyte/sporophyte) does not entail a role for ploidy in determining the identity of the two organizational forms of the same organism, gametophyte and sporophyte. This concurs with the observation that there are deep homologies in the genetic control of haploid-to-diploid transition across distantly related eukaryote taxa, regardless of the disparity in the significance of the two phases among these lineages. Thus, despite the peculiarities of this kind of cycles, not differently from other cycles with multiple developmental pathways, control and regulation of gametophyte and sporophyte development rest mainly on a differential transcriptome, including both identity and expression level of a set of key genes, integrated by different forms of epigenetic regulation.

#### 3.3.2 Metagenetic cycles

A metagenetic cycle is a multigenerational cycle in which exclusively asexual generations alternate with a sexual generation, represented by distinct organizational forms. Metagenetic cycles are distinguished from other multigenerational cycles by the fact that there is at least one obligate asexual generation, morphologically and physiologically distinguishable from the sexual generation with which it alternates. Depending on the organism, one or more organizational forms can include multiple asexual generations through which the same organizational form is repeated.

Metagenetic cycles are found in many metazoans, including cnidarians, dicyemids, digeneans, cestodes, polychaetes, cycliophorans and tunicates.

Cnidarians of the clade Medusozoa have a metagenetic life cycle with alternating generations of sedentary polyp and pelagic medusa, or a derivative form of this cycle (Fautin, 1992). Asexual reproduction may lead to the production of (i) medusae from polyps, but also (ii) medusae from medusae (only in Hydrozoa) and (iii) polyps from polyps. There are different modes (mechanisms) of asexual reproduction, and thus, possibly, of development. In many cnidarians the generation of new polyps from a parent polyp is not followed by the detachment of the new polyps; this is how colonies develop, another form of multiple development (see Section 3.3.4). Among derived cnidarian life cycles, all sorts of multiple developmental pathways can be found (Fusco and Minelli, 2019).

**FIGURE 5:**
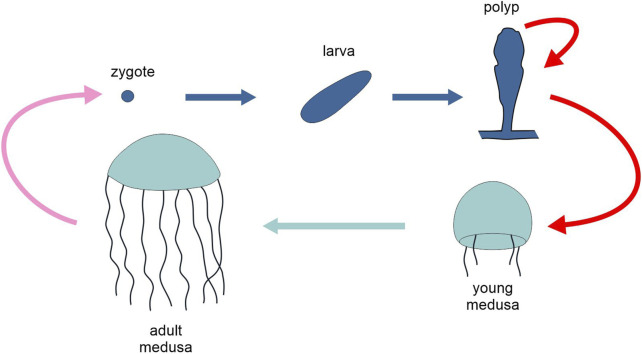
Schematic representation of the life cycle of the cnidarian *Clytia hemisphaerica*, highlighting the two generations, polyp and medusa (different tones of blue). Pink arrow, sexual reproduction; red arrows, asexual reproduction; light and dark blue arrows, development. Figure not to scale.

The trascriptomics of the “typical” polyp-medusa alternation has been investigated in a few model species. In the hydrozoan *Clytia hemisphaerica* (Figure 5), medusa, planula larva and polyp are characterized by distinct transcriptome profiles, with polyp and medusa transcriptomes closer to each other than either is to the planula stage, which is separated from the adult polyp stage by a drastic metamorphosis. Both adult stages express more genes and with a more complex pattern than the planula. Focussing on the expression of transcription factors, the majority of those that are stage-specific are found in the medusa (34), one third of which plausibly sex-specific, while a smaller number (12) are polyp-specific. The transcription factors that are expressed at either or both the polyp and medusa stages, but not in other stages (62), amount at a 12% of the total. Medusa stage-specific transcription factors include several bilaterian orthologues, associate with diverse neurosensory structures (Leclere et al., 2019).

The transcriptomes in the passage from polyps to juvenile medusae through strobilation has been characterized by RNA sequencing in the scyphozoan jellyfish *Rhopilema esculentum* (Ge et al., 2018). Strobilation is elicited by specific environmental signals, and 7090 (out of ∼96,000) assembled transcripts are differentially expressed among the different stages.

Moving to the platyhelminths, the life cycles of parasitic flatworms are very diverse, often complex and still of uncertain interpretation. In the Digenea (see Galaktionov and Dobrovolskij, 2003), two “phases” are recognized, one with sexual reproduction (*marita*, normally considered as one generation), one with asexual reproduction (actually, often interpreted as parthenogenetic, hence the name *parthenita*), including multiple generations and organizational forms. Although in these parasitic flatworms there is no such thing as a “typical” life cycle, in many cases, a hermaphrodite adult (gonochoric in *Schistosoma*), called the *marita* stage, lives as a vertebrate parasite, and produces eggs that are fertilized and released into the environment, from which a free-living aquatic larva develops. This tiny larva, the *miracidium*, can infect a mollusc. In the body of the latter, the miracidium develops into a *mother sporocyst*. This reproduces asexually (or, according to some interpretations, by parthenogenesis), generating a second generation of sporocysts (*daughter sporocysts*) or, depending on the species, a first generation of *rediae*. Both daughter sporocysts and rediae can reproduce asexually (or, according to some interpretations, by parthenogenesis). Sooner or later, daughter sporocysts (or rediae) generate a new type of larva, the *cercaria*, which sometimes develops into a non-mobile *metacercaria* before maturing into a marita, finally closing the cycle.

That the reproduction of sporocysts and rediae is actually by parthenogenesis, rather than asexual, is a still open question (see Nesterenko et al., 2022); in the former case this cycle should be described as heterogonic rather than metagenetic (see next Section). But it is possible that uniparental reproduction follows different cytogenetic modes in different species, or between different reproductive events within the same cycle. Redia, cercaria, metacercaria and adult marita are very diverse life stage along these metagenetic cycles and gene expression research has concentrated on differences among them.

Nesterenko et al. (2020) studied the transcriptomes of rediae, cercariae and adult worms of *Psilotrema simillimum* and *Sphaeridiotrema pseudoglobulus*. In *P. simillimum* more than 60% of analysed genes were expressed in all stages, whereas in *S. pseudoglobulus* less than 40% of genes showed such a generalised expression pattern. About 36% of genes were preferentially expressed in one of the three stages of *P. simillimum* and 66% in *S. pseudoglobulus*. In both species, a sizable change in the levels of expression for most of the genes was observed, so that the molecular signature of a given stage is not only characterized by a specific set of expressed genes, but also by specific levels of their expression.

Metagenetic cycles are also found in the Cestoda. The life cycle of some tapeworms is distinctly bigenerational, due to the intercalation in the sexual cycle of a stage (such as the hydatid cyst of *Echinococcus granulosus* or the multilocular cyst of *Echinococcus multilocularis*) from which a number, sometimes very high, of individuals capable of reaching sexual maturity is produced asexually. In *E. granulosus*, hydatid cysts produce protoscoleces (a juvenile stage of the worm), which may remain in an inactive state within the body of the intermediate host for years. When a definitive host swallows an infected part of the latter, the protoscoleces may be released from the cysts and develop into adult worms in the definitive host’s intestine. However, if a hydatid cyst ruptures within an intermediate host, each protoscolex is capable of developing into a secondary hydatid cyst. Bai et al. (2020) studied the global transcriptome and microRNAome of this worm and found that 963 genes and 31 miRNAs are expressed differentially during protoscolex development into an adult worm, and similar numbers of 972 genes and 27 miRNAs are differentially expressed during protoscolex development into a cyst. This ‘bi-directional development’, as the authors call it, evidently includes features of a cycle with developmental options (Section 3.1).

Although generally not described as an example of metagenesis, some benthic polychaetes belonging to the family Syllidae exhibit a unique life cycle that can be described as such. Benthic syllid species produce “epitokes,” i.e., sexually mature worms, which swim and spawn gametes in the reproductive season. In the plesiomorphic reproductive mode (called “epigamy”), observed in some species, the entire body of a benthic individual develops into an epitokous morph (“linear developmental complexity,” see Introduction; not an instance of metagenesis). In contrast, in some other species that reproduce by “schizogamy” (also called “stolonization”), only the posterior part of a mature individual becomes an epitokous unit (termed a “stolon”) carrying gametes, which detaches from the benthic individual for spawning. After stolon detachment, the parental benthic individual, termed the “stock,” regenerates its posterior segments so that it can repeatedly generate stolons. This can be described as a metagenetic cycle, with stolonization as a form of asexual (iteroparous) reproduction for the benthic organizational form. In fact, the stolons that are generated are fully independent (offspring) individuals, with their own sensory organs, such as eyes and antennae, well-developed muscular and nervous systems and swimming appendages. They swim in response to the presence of opposite-sex stolons and in several syllid species a brain-like nerve structure is newly formed. In *Megasyllis nipponica*, Nakamura et al. (2023) showed that the formation of a stolon begins with gonad maturation, followed by the development of a cerebral ganglion and by stolon-specific structures, such as stolon eyes and chaetae. As for gene expression profiles, in the posterior of the body, genes for gonadal development are upregulated, as well as hormone-related and head-determination genes, while Hox genes, involved in the regional specification along the anterior–posterior axis, show no significant temporal expression changes.

The cycliophorans, a phylum of microscopic marine animals, have a very complex metagenetic life cycle, as well (Wanninger and Neves, 2015). Although no information on developmental regulation is available, nonetheless they are worth mentioning here because, interestingly, and to our knowledge uniquely among the metazoans, their cycle is two-generational through the female and three-generational through the male, offering a remarkable case of sexual dimorphism in the structure of the alternation of generations of the life cycle.

Life cycle with alternation of sexual and asexual generations are very common in tunicates as well, with a high disparity in the mechanisms of asexual reproduction (Brown and Swalla, 2012). However, since these are in general associated with an alternation of solitary and colonial organization, we will treat them in Section 3.3.4.

Metagenetic cycles beautifully illustrate the fact that the different forms of developmental complexity treated in this review do not form disjoint categories, presenting instead wide regions of overlap. The case of parasitic flatworms shows an uncertain boundary with heterogonic cycles, and when asexual reproduction leads to the formation of colonies, it is the boundary with the cycles alternating solitary and colonial organization which demonstrates a certain fuzziness, as exemplified by the case of colonial tunicates mentioned above. Furthermore, the ‘bi-directional development’ of the cestode *E. granulosus* exemplifies a case where a metagenetic cycle (sequential developmental complexity) presents traits of a cycle with developmental options (parallel developmental complexity). Finally, the distinction between multigenerational metagenetic life cycles and monogenerational life cycles with a biphasic development marked by a metamorphosis rests on the interpretation of a key passage in the life cycle, either as reproduction or development, respectively. In a typical cnidarian life cycle, the transition from polyp to medusa is interpreted as a reproductive event separating two distinct generations. Conversely, in the typical life cycle of sea stars, the transition from larva to juvenile is interpreted as the metamorphosis of the same individual. However, the distinction between metagenesis and metamorphosis is not always unambiguous (Minelli, 2009; Fusco and Minelli, 2019). In cubozoan cnidarians, the polyp disappears when literally transforming into a medusa. Should this count as a reproductive or a developmental event? On the opposite side, in the metamorphosis of many marine invertebrates, most of the larval body is discarded and the young derives from a small number of founding (set-aside) cells. In the sea star *Luidia sarsii* the larva can even continue to swim for months after the juvenile that originated from it has detached (Williamson, 2006). Should this count as a developmental or a reproductive event? The matter is generally resolved by taxon-specific tradition, but this should not obscure the connections among these only apparently completely separate types of cycle.

#### 3.3.3 Heterogonic cycles

**FIGURE 6:**
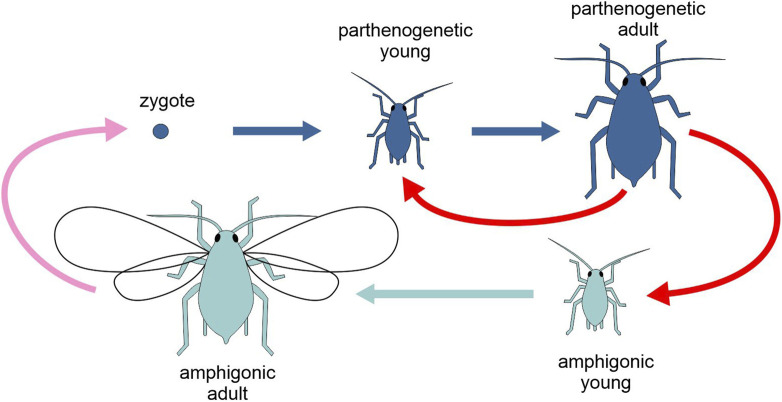
Schematic representation of the life cycle of the aphid *Acyrthosiphon pisum*, highlighting the two types of generation, amphigonic and parthenogenetic (different tones of blue). Pink arrow, amphigonic reproduction; red arrows, parthenogenetic reproduction; light and dark blue arrows, development. Figure not to scale.

In some eukaryotes, amphigonic reproduction alternates regularly with parthenogenesis. These multigenerational cycles, called heterogonic cycles, are found in some species of parasitic nematodes and in most monogonont rotifers, cladoceran crustaceans and aphids (Figure 6). In these animals, the transition from parthenogenesis to amphigonic reproduction depends on the interpretation of specific environmental cues, like day length or population density.

Not all heterogonic cycles involve multiple developmental pathways, if, for instance, the distinct gametogeneses that lead to a reduced (haploid) or to an unreduced (diploid) egg converge, upon fertilisation in the former case, without fertilisation in the latter case, to a similar zygotic stage, as it occurs in oviparous aphids. But there are situations where the products of amphigonic and parthenogenetic reproduction involve distinct developmental pathways. This is the case, for example, of the so-called viviparous aphids (Aphididae).

In viviparous aphids, within the same species, embryogenesis takes place within the shell of a laid egg in the generation originating from amphigonic reproduction (“oviparous development”), while the embryo develops nourished by the mother haemolymph within the ovary, with the mother giving live birth progeny, in generations deriving from parthenogenetic reproduction (“viviparous development”). In some viviparous aphids, a parthenogenetic female carries in her body her developing daughters, and within these their own daughters (granddaughters of the former), like Russian dolls. These telescoped generations see the shortest generation time among insects, up to less than 5 days, as in the aphid *Rhopalosiphum padi.*

Lin et al. (2022) reviewed similarities and difference between the two types of embryonic development in the pea aphid *Acyrthosiphon pisum*. Viviparous embryogenesis is an order of magnitude faster than oviparous embryogenesis, approximately 10 days compared to 100 days, and viviparous eggs are also significantly smaller at the time of key patterning events in early development, with nearly a fivefold difference in egg length. In both viviparous and oviparous embryos, a presumptive germ plasm (cytoplasm containing germline determinants, like maternal Vasa protein) is localised in the posterior region of the eggs, to be later incorporated into the newly formed germ cells. However, the timing of appearance of the germ plasm is significantly brought forward in viviparous embryos with respect to oviparous embryos, with a possible involvement as a regulator of posterior development. As for anterior-posterior axis determination, there are marked differences in the expression domain of key developmental genes (*hunchback*, *orthodenticle*, and *caudal*) between oviparous and viviparous development, both during oogenesis and early embryogenesis. Oviparous development is of the short-germ band type, like in grasshoppers, where gastrulation starts with just the head and the most anterior trunk segments, and new abdominal segments are formed from a growth zone located in the posterior of the embryo (Chipman, 2015). In contrast, in viviparous development, although abdominal segments are gradually formed during embryogenesis, the germband occupies most of the egg length, similarly to that of long-germ insects, like *Drosophila*.

In the same aphid model species, *A. pisum,* Matsuda et al. (2020) investigated gene expression involved in the switch between the two reproductive modes. In many aphid species, the alternation of generations is regulated by photoperiod: oviparous sexual females and males are produced under short days, in autumn, while viviparous parthenogenetic females are produced under long days in spring and summer. However, the system needs a second mechanism of regulation, called “seasonal timer,” which suppresses the sensitivity to photoperiod during the first spring generations, in early spring, when days are still relatively short, and females reproduce nonetheless exclusively by parthenogenesis. Matsuda et al. (2020) found that under short days, at the time of the switch to sexual reproduction, aphids with an expired (no longer operative) seasonal timer show a higher expression level in hundreds of genes than those with a still operative seasonal timer. Under the same conditions, they also observed a higher frequency of epigenetic modifications, at the level of histones and small non-coding RNA pathways, in aphids with an expired seasonal timer, suggesting that these epigenetic regulations of gene expression might be involved in the mechanism of maternal switching from parthenogenetic to amphigonic offspring.

As in the case of certain cycles with parallel developmental complexity (Section 3.1), the switch between alternative developmental pathways in viviparous aphids is under maternal control, another feature that cross-sects our classification of life cycles with multiple developmental pathways.

#### 3.3.4 Alternation of solitary and colonial generations

Multigenerational cycles of some multicellular organisms are characterized by a phase of aggregation among the individuals that are generated, usually indicated as a colony. When the colony presents a species-specific form and/or a certain level of integration of the single individuals and/or their divergent specialization, the solitary individual and the colony as a whole can be regarded as two different organizational forms of the same organism. In these cases, the development of the solitary individual is a different kind of development with respect to that of a colony, although some biologists would not call the latter process “development.”

In many marine invertebrates, the planktonic larva of a founding individual finds an appropriate place to settle, metamorphoses and begins a sedentary adult phase. At a certain point it begins to reproduce asexually, typically by budding, generating a progeny of individuals often called zooids, which in turn will continue to reproduce asexually, but remaining somehow connected, thus producing the colony. The primary zooid (the founding individual of the colony) may be morphologically similar to the secondary zooids that originate from it (as in the red coral, *Corallium rubrum*), but it may also differ considerably (as in sea pens corals of the genus *Pennatula*). Sexually competent secondary zooids produce gametes that fertilize to form zygotes that will develop into the solitary planktonic larvae of the following generation. Unlike metagenetic cycles (Section 3.3.2), here the two organizational forms, the solitary and the colonial, do not obligatorily exhibit a different reproductive mode (Fusco and Minelli, 2019), since primary and secondary zooids can both reproduce asexually, while the latter can switch to sexual reproduction. However, the cycle of many colonial hydrozoans can be assigned to both kinds of cycles, solitary-colonial and metagenetic.

There are different ways in which the development of the solitary individual and the colony that derives from it may differ: (i) colony development relies on asexual propagation of the members of the colony through species-specific forms of “non-embryonic development” (Alié et al., 2018), that is, a developmental process that does not start from a zygote; (ii) the colony can present species-specific morphogenesis during growth, producing a colonial phenotype that can be largely independent of the morphology of the individual members of the colony and sensitive to a different array of environmental factors; (iii) the members of the colony can present some level of differentiation/specialization (polymorphism), a form of developmental plasticity otherwise not expressed at the level of the solitary individual.

**FIGURE 7:**
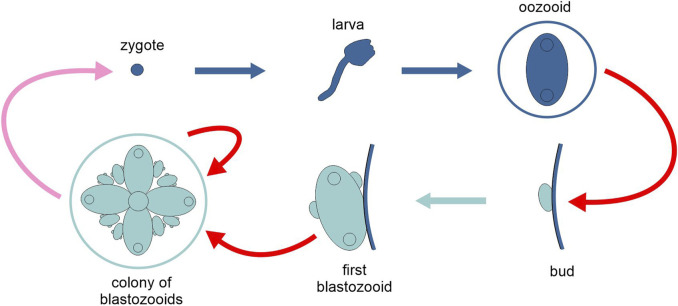
Schematic representation of the life cycle of the tunicate *Botryllus schlosseri*, highlighting the two types of generation, solitary and colonial (different tones of blue). Pink arrow, sexual reproduction; red arrows, asexual reproduction; light and dark blue arrows, development. Figure not to scale.

Many aquatic invertebrates form permanent colonies through asexual reproduction. These include several cnidarian lineages (like most corals), pterobranchs, tunicates (Figure 7), bryozoans and kamptozoans (Hiebert et al., 2021). Depending on the species, zooids can (i) remain connected anatomically and physiologically through living extensions (“compound colonies,” e.g., the ascidian *Botryllus schlosseri*, where zooids share a vascular network), (ii) secrete material to embed the individuals (“social colonies,” e.g., most Polyzoinae ascidians, where zooids are only embedded in a common tunica), or (iii) simply stay nearby each other with no reciprocal connection (“clonal aggregates,” e.g., catenulid flatworms of the genus *Alaurina*). Zooids can be either identical or morphologically differentiated, so that functions like feeding or reproduction can be distributed among distinct units of the colony (Alié et al., 2018).

Mechanisms and control of these dual developmental trajectories (following sexual and asexual reproduction, respectively) in a single species have been intensively studied in tunicates. In colonial ascidians, the founding individual of a colony, derived from sexual reproduction and embryogenesis, is called the oozooid, which through a number of asexual budding cycles generates multiple blastozooids (blastogenesis). Oozooids and blastozooids show certain anatomical differences, for instance at the level of the pharyngeal slits, although the general architecture of the body is maintained and many developmental genes expressed during embryogenesis are likewise expressed during the formation of the bud. For example, in *B. schlosseri*, genes expressed in two sub-domains of the metamorphosing larva (orthologs of transcription factors of the vertebrate placode gene network, like *Six*, *Eya* and *FoxI*) are likewise expressed in corresponding regions of the developing bud (Stolfi and Brown, 2015). The phylogeny of tunicates suggests that asexual reproduction with the production of colonies has evolved independently multiple times within the group: this is also supported by the fact that budding mechanisms of different species involve nonhomologous tissues and cells (Alié et al., 2021).

In many tunicates, whole-body regeneration (WBR), i.e., the ability to regenerate the entire body from a small number of cells, is a characteristic of their life cycle (“propagative budding”), but it can also be activated following extensive injury, in which case the WBR process is referred to as ”survival budding” (Ricci et al., 2022). The phylogenetic distribution and the diversity of the cellular details of this phenomenon suggest that WBR capacity through budding evolved multiple times among the tunicates (Alié et al., 2018; Alié et al., 2021; Nydam, 2020).

In bryozoans, the founding zooid of a colony undergoes a radical metamorphosis after settling, where most of the larval tissues are destroyed, to be replaced by those of the adult. This process is not part of the development of the secondary zooids, which are generated through budding. Fuchs et al. (2011) investigated the expression domain of 13 developmental genes in the larval stage of the gymnolaemate bryozoan *Bugula neritina* and found that most of these genes are expressed in areas of the larval blastemic tissues that contribute to the definitive adult body. Only two of the 13 genes were exclusively expressed in larval tissues that are discarded at metamorphosis. As in other colonial marine invertebrates, bryozoan colony persistence depends on a turnover of zooids: new zooids are generated by budding, while old zooids degenerate. Kvach et al. (2025) performed extensive RNA sequencing during polypide development in the freshwater bryozoan *Cristatella mucedo*, from bud, to mature stages to degeneration, showing that colony development depends on the expression of conservative stemness markers, in developing buds and juvenile zooids, while zooid degeneration involves autophagy and other types of programmed cell death.

Another feature of colony development is within-colony zooid polymorphism. Changes in gene expression that control bryozoan zooid polymorphism are currently unknown (Schack et al., 2019). However, the same question has been investigated in cnidarians, studying polyp polymorphism in colonies of the hydrozoan *Hydractinia*. This is under the control of a panel of differentially expressed functional, structural, and patterning genes. Out of an assembly of ∼65,000 transcripts generated using RNA-seq libraries, constructed from different kinds of polyp (feeding, reproductive, defensive) of *Hydractinia symbiolongicarpus*, ∼7000 transcripts were differentially expressed in a way specific for the different kinds of polyp (Sanders et al., 2014). In the same species, Cartwright et al. (1999) documented that the development and maintenance of specialized polyps is controlled by differential levels of expression of the Gsx Parahox gene *Cnox-2*. Cartwright et al. (2006) found that this gene plays prominent, but distinct roles in both the ontogeny of the polyp and the ontogeny of the colony as a whole. In the polyp, Cnox-2 seems to suppress the development of oral structures at the aboral pole of the body column, following the establishment of the mature axis. In the patterning of the colony, Cnox-2 appears to specify those locations of the tube-like structures connecting the polyps (stolons, or rhizomes) where tip and polyp rudiments will form. Indeed, the neat expression boundary between the base of the polyp and the stolon from which it emerges suggests that Cnox-2 is a marker for polyp–stolon boundaries. In the same species, through single cell transcriptomic profiling, Salamanca-Díaz et al. (2024) identified cell type-specific transcription factors and gene networks across the different parts of the colony. They found that different kinds of polyps are primarily characterised by distinct combinations of cell types, rather than by polyp-kind-specific cell types. However, they also recognised a previously unidentified stolon-specific cell type, which expresses enzymes related to biomineralization and chitin synthesis.

Alternation of solitary and colonial generations has evolved independently multiple times, and this is reflected in a significant diversity in several features of the associated developmental processes, as observed, for instance, in the involvement of nonhomologous tissues and cells in budding in taxa that are relatively close relatives. A common feature seems nonetheless to be that many developmental genes expressed during embryogenesis of the solitary individual are likewise expressed during the growth of the colony. However, there are marked differences between the two developmental processes, that of the solitary founder of the colony and that of the colony itself. Despite the development of the solitary form can constrain that of the colony, as suggested for the bryozoans, and the large regions of overlap between the development of the founding individual and all its descendant modules of the colony, emergent morphogenetic process at the level of the colony take the phenotypes to another level of organization, with respect to anatomy, morphology and physiology, involving different forms of plasticity, sensitive to different environmental factors with respect to individual development.

#### 3.3.5 Alternation of unicellular and multicellular generations

Some eukaryotes that can reproduce both as unicellulars and multicellular aggregates are generally considered organisms with an organization at the edge between colonial unicellularity and true multicellularity. The individual-colony alternation, presented in the previous section, is translated here in an alternation of unicellular and multicellular organizational forms of the same organism. This is paradigmatic when the aggregation presents a species-specific morphology (and accordingly, morphogenesis) and the cells that compose it show high levels of integration and differentiation. Here, as well, there are at least two different kinds of development: that of the solitary unicellular individual and that of the colonial aggregate.

Multicellularity has evolved multiple times in the history of life. Adopting a wide concept of multicellularity, Lamža (2023) identified 45 independent multicellular lineages in eukaryotes. These can be grouped into different types, depending on the origin of the multicellular aggregate, e.g., by the division of a single founding cell (*clonal multicellularity*) or by the gathering of multiple separate cells (*aggregative multicellularity*), and on the structure of the aggregate, from septate multinucleated thalli to pseudoplasmodial forms, to bodies made of multiple fully compartmentalised cells.

Although all these taxa are potentially of interest for the evolution of multicellularity, not all of them present the kind of alternation of generations discussed in this section. Here, we consider only the protists with an aggregative phase in their life cycle, either obligate or facultative, which can reproduce both in the solitary and the aggregate phase. This leaves out “frankly” multicellular organisms, like animals, land plants, many fungi, brown and red algae, which, as a norm, do not reproduce as a single cell. To this list of excluded taxa, one should add some other lineages that are intensively studied in the context of the evolution of multicellularity, but do not present alternation of unicellular and multicellular generations, like the Volvocinae green algae, which do not reproduce as solitary vegetative cells, and the Myxogastrida, or plasmodial slime moulds, which do not reproduce in the amoeboid solitary phase.

**FIGURE 8:**
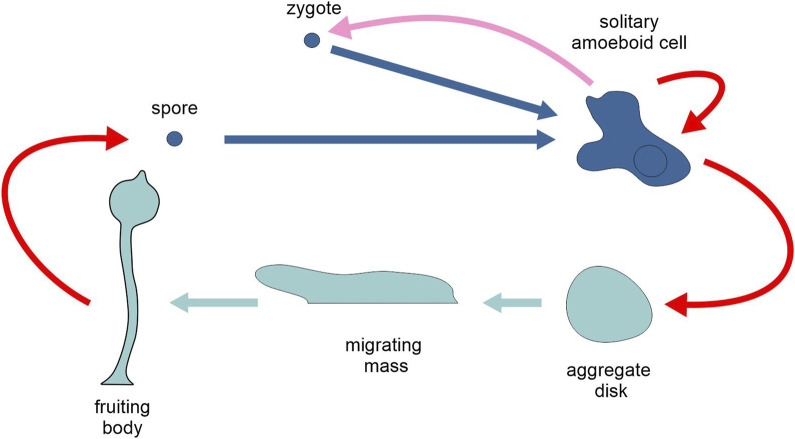
Schematic representation of the life cycle of the cellular slime mould *Dictyostelium discoideum*, highlighting the two types of generation, unicellular and multicellular (different tones of blue). Pink arrow, sexual reproduction; red arrows, asexual reproduction; light and dark blue arrows, development. Figure not to scale.

The most intensively studied group of organisms with an alternation of unicellular and multicellular generations are the cellular slime moulds (Dictyostelea), which present aggregative multicellularity (Figure 8). In dictyosteliids, there is an asexual cycle where the trophic phase is represented by haploid solitary amoeboid cells that feed on soil bacteria and reproduce asexually by binary fission. In *Dictyostelium discoideum*, when food is scarce, many cells start aggregating, reciprocally exchanging chemical signals based on cyclic adenosine monophosphate, forming ripples and streams first and then an approximately discoidal mass. The disc turns into a migrating mass which after a short wandering stops and turns into a pedunculated fruiting body where some of the cells become resistant spores that are released and dispersed into the environment. Under favourable conditions, the wall of the spore breaks apart, allowing the amoeboid cells to return to the trophic phase and to asexual reproduction. In this asexual cycle, reproduction occurs by binary fission during the unicellular phase and by spore production during the multicellular phase. Free amoebae can also switch to a sexual cycle. Here two amoebae undergo syngamy and form a zygote that grows by attracting and engulfing solitary individuals of the same species (cannibalism). This giant zygote acquires a wall and becomes resistant. It undergoes meiosis, followed by several mitotic cycles, so that from a zygote several amoeboid haploid cells are issued, ready to resume the trophic phase.

Many cellular and molecular events critical to development have been elucidated through genetic and chemical experiments on *D. discoideum*, and the developmentally regulated, protein-coding transcriptome has been characterized extensively (e.g., Rosengarten et al., 2015), as well as the expression of long noncoding RNAs (ncRNAs) (Rosengarten et al., 2017). Developmental events and gene expression are highly correlated; nonetheless, transcriptional and morphological changes are not always precisely coordinated in time. For instance, some of the major changes in gene expression occur before the start of morphogenesis and aggregation, whereas the profound morphological changes that go along with the production of the fruiting body are associated with only a few transcriptional changes (Rosengarten et al., 2015).

In a time-series RNA-sequencing analyses of both wild type and mutant *D. discoideum* strains, Katoh-Kurasawa et al. (2021) identified 1371 genes that undergo sharp changes in the level of expression during eight developmental-stage transitions, with a tendency to a decreasing number of genes involved in phase transition as development progresses.

A very interesting case of study in clonal multicellularity is offered by the choanoflagellate *Salpingoeca rosetta*. As a response to specific environmental cues, this protist can differentiate into at least five distinct morphotypes: three unicellular forms (slow swimmers, fast swimmers, and thecate cells) and two colonial forms (rosettes and chains) (Dayel et al., 2011). Thecate solitary cells are sessile and can produce solitary swimming cells either through cell division or by abandoning the theca. Solitary swimming cells can divide and separate completely to produce solitary daughter cells, or remain attached after division, producing colonies of either type. Colonies can divide to produce daughter colonies. Interestingly, the development of rosette colonies is induced by prey bacteria, through the action of a specific sulfonolipid produced by these prokaryotes (Alegado et al., 2012).

Transcriptome studies showed that conserved genes for cell division contribute to *S. rosetta* colony formation, indicating that the initiation of colony development may rely on genes shared with metazoans, while later stages of colony formation are apparently regulated by genes unique to *S. rosetta* (Fairclough et al., 2013).

Fumagalli et al. (2023) analyse the same transcriptomic data to identify a core of genes associated with the formation of multicellular colonies. There are about 2800 differentially expressed genes between the different morphotypes, with the thecate type showing the most pronounced differences with respect to all the other types. There is a core of 340 genes whose expression is at least four-fold increased or decreased in colony cell state with respect to solitary states, which includes genes for putative collagen and microtubule-associated proteins.

Beyond the unicellular-multicellular alternation, the cycle of *S. rosetta* includes other kinds of developmental complexity, in the alternative reproductive/developmental pathways among the solitary forms. Since single cell individuals can change their phenotype either with or without division (reproduction), the same transformation (e.g., from thecate to swimming cell) may count as a change of generation (sequential developmental complexity, in the generic sense of an alternation of generations with different organizational form), or as a segment of an optional ontogenetic pathway for the same individual (parallel developmental complexity; Section 3.1), which bring into light another blurry boundary between different kinds of developmental complexity.

Finally, although the phenomena of phenotypic plasticity indicated as ecomorphosis and cyclomorphosis (distinct morphs of the same organism are expressed under unfavourable environmental conditions, or cyclically at different times of the year, respectively) are not included in our review (see Section 3.2), we cannot miss to mention here the case of the planktonic green algae of the genus *Scenedesmus*, where plasticity is characteristically associated with a unicellular-multicellular alternation of generations. These algae present solitary and colonial forms (coenobia) of different types, and the transition through different forms can occur both cyclically and non-cyclically, as a response to variation in population density and available resources (Egan and Trainor, 1991).

In evolutionary biology, there is a growing interest in the evolution of multicellularity, but the connections between the emergence of multicellularity and the evolution of developmentally complex life cycles are still to be explored. The cases presented here show several points in common between these unicellular-multicellular cycles and other cycles with multiple development discussed above, for example, with respect to the role of differential gene regulation through differential transcription and other epigenetic regulative processes.

## 4 Conclusion

We have presented here a reasoned classification of developmentally complex life cycles to facilitate comparison between taxa and cycles at the large scale of all eukaryotes and to bring to light major structural differences among them. However, this should not conceal the extensive interconnections among phenomena that for practical reasons are classified here under separate headings. This is testified by the numerous ‘grey zones’ and ‘borderline cases’ mentioned throughout this review, and by the similarities between developmental regulation and control through the alternative or sequential segments of a life cycle and through the different segments of the same ontogeny (linear developmental complexity) that we left out of the main discussion. For instance, transcriptome analyses throughout *Drosophila* development including embryonic, larval, pupal, and adult stages, revealed that a large fraction of the fly’s genes undergo significant expression level changes across development. Different stages are characterized by unique gene expression profiles. Substantial transcriptome remodelling occurs during the larval-pupal and pupal-adult transitions, reflecting the extensive physiological changes during metamorphosis (Ozerova and Gelfand, 2022). Furthermore, the developmental process is marked by pervasive alternative splicing, with different gene isoforms often showing stage-specific expression patterns (Yeoh et al., 2019). The identification of non-coding RNAs with stage-specific expression profiles (Chen et al., 2016) adds another element of similarity with other types of developmental complexity (Brown et al., 2014).

In this article we have not touched the vast subject of the evolution of life cycles, but a few words are in order here. Life cycle evolution is a challenging subject of study (Fusco, 2019 and references therein), but most of the relevant literature is taxonomically restricted and a general theoretical treatment is still lacking. By acknowledging the life cycle as a “unit of evolution” (Minelli, 2009), it is possible to contemplate the possible sources of selectable variation more inclusively (Fusco, 2019). Many kinds of evolutionary changes are modifications of specific features of the structure of the life cycle, such as its articulation into one or more organizational forms, or the specific mode of reproduction of one of these to the next. For instance, a new organizational form (the carposporophyte) has been added to the primitively biphasic cycle of many red algae (e.g., *Polysiphonia*) (Yang et al., 2016), while one organizational form (the gametophyte) of a primitive haplodiplontic cycle has been suppressed in the cycle of some brown algae (e.g., *Fucus*) that have a monogenerational diplontic cycle (Heesch et al., 2021). Another point is that the complexity of the biological cycle and the morphological complexity of the organism are largely independent. Developmental complexity has been frequently increased, without any obvious consequence for the morphological complexity of the pre-existing stages, adult included, by addition of a new intercalary stage, such as the pupa of holometabolous insects, and novel first larval stages, such as the triungulin of blister beetles and other hypermetabolous insects (Minelli et al., 2006). On the opposite, morphological simplification is not necessarily coupled with decreasing developmental complexity, as witness the Myxozoa, now recognised as morphologically highly simplified forms of Cnidaria, which nevertheless retain considerable life-cycle complexity (Siddall et al., 1995).

But even restricting our focus to proximate-cause questions, as we have done, the quantity and disparity of biological phenomena involved is vast. At a molecular level, these include regulation of gene transcription, alternative splicing, epigenetic modifications, long and short noncoding RNA regulation and transposable element activity, while at cellular level and beyond we found cell differentiation and dedifferentiation, stem-cellness regulation, morphogenesis and morphallaxis, developmental repatterning, heterochrony, sensitivity to environmental factors and maternal control. This variety is mirrored by the arsenal of methodologies adopted to investigate regulation and control of the multiple developmental pathways in the same organism: QTL analysis, gene expression profiling through RNA-seq, gene expression localisation by *in situ* hybridization, analysis of the pattern of methylation. Future investigations on the disparity of complex life cycles and their evolution will certainly benefit by the spreading of new technologies, e.g., in epigenetic analysis and single cell RNA sequencing, but also dedicated theoretical work is in order, particularly in developing comprehensive models that integrate molecular, developmental, and evolutionary perspectives to better understand the mechanisms driving the diversity and plasticity of life cycle strategies across the tree of life.
